# Evidence of Lumpy Skin Disease Virus Transmission from Subclinically Infected Cattle by *Stomoxys calcitrans*

**DOI:** 10.3390/v15061285

**Published:** 2023-05-30

**Authors:** Andy Haegeman, Charlotte Sohier, Laurent Mostin, Ilse De Leeuw, Willem Van Campe, Wannes Philips, Nick De Regge, Kris De Clercq

**Affiliations:** 1 Sciensano, Infectious Diseases in Animals, Exotic and Vector-Borne Diseases, Groeselenberg 99, B-1180 Brussels, Belgium; charlotte.sohier@sciensano.be (C.S.); ilse.deleeuw@sciensano.be (I.D.L.); nick.deregge@sciensano.be (N.D.R.);; 2 Sciensano, Experimental Center Machelen, Kerklaan 68, B-1830 Machelen, Belgium; laurent.mostin@sciensano.be (L.M.);; 3 EURL for Diseases Caused by Capripox Viruses, Sciensano, Groeselenberg 99, B-1180 Brussels, Belgium; wannes.philips@sciensano.be

**Keywords:** *capripox virus*, vector transmission, subclinical infection, stable fly

## Abstract

Lumpy skin disease virus (LSDV) is a vector-transmitted capripox virus that causes disease in cattle. *Stomoxys calcitrans* flies are considered to be important vectors as they are able to transmit viruses from cattle with the typical LSDV skin nodules to naive cattle. No conclusive data are, however, available concerning the role of subclinically or preclinically infected cattle in virus transmission. Therefore, an in vivo transmission study with 13 donors, experimentally inoculated with LSDV, and 13 naïve acceptor bulls was performed whereby *S. calcitrans* flies were fed on either subclinical- or preclinical-infected donor animals. Transmission of LSDV from subclinical donors showing proof of productive virus replication but without formation of skin nodules was demonstrated in two out of five acceptor animals, while no transmission was seen from preclinical donors that developed nodules after *Stomoxys calcitrans* flies had fed. Interestingly, one of the acceptor animals which became infected developed a subclinical form of the disease. Our results show that subclinical animals can contribute to virus transmission. Therefore, stamping out only clinically diseased LSDV-infected cattle could be insufficient to completely halt the spread and control of the disease.

## 1. Introduction

Lumpy skin disease (LSD) is a viral cattle disease caused by the lumpy skin disease virus (LSDV), which belongs to the *Capripoxvirus genus*, subfamily *Chordopoxvirinae*, family Poxviridae. The World Organization for Animal Health (WOAH) categorizes LSD as a notifiable disease because of the substantial socio-economic impact of an outbreak [1]. LSD is endemic in southern, central, eastern and western Africa. Prior to 2012, only sporadic LSD outbreaks were reported in the Middle East region. However, since then, LSD spread to the Middle East, south-Eastern Europe, Russia and recently, many Southeast Asian countries.

Some of the first clinical symptoms observed after an LSDV infection are fever (with peaks of 40/41 °C), inappetence, and swelling of subscapular and precrural lymph nodes become noticeably enlarged. Shortly after the onset of fever, the skin nodules start to develop. The amount and localization can vary from a few skin nodules to multiple lesions covering the entire animal. In severely affected animals, ulcerative lesions appear in the mucous membranes of the eyes and oral/nasal cavities, causing excessive lachrymation, salivation and nasal discharge. Pox lesions may also be present in the pharynx, larynx, trachea, lungs and throughout the alimentary tract. Temporary or permanent infertility occurs among infected cows and bulls [1]. The disease is more severe in cows at the peak of lactation and causes a sharp drop in milk yield because of high fever caused by the viral infection itself and secondary bacterial mastitis. The incubation period in natural outbreaks is estimated to be 2–5 weeks [2]. During LSDV outbreaks, reported morbidity rates vary between 3 and 85% depending on the immune status of the hosts and the abundance of arthropod vectors that mechanically transmit the virus. The mortality rate is generally low (1–3%) but may sometimes reach 40% [3]. In addition to animals displaying the typical skin nodules, LSDV can also cause a subclinical form of infection which has been reported in the field [1]. Those animals do not develop skin nodules, but proof of productive virus replication can be found through laboratory analyses.

LSDV is a vector-borne disease contributing to the recent rapid geographic spread of the virus (3). Multiple blood-sucking arthropods, such as biting flies, such as stable flies, midges, horse flies and ticks, have been suggested to play a potential role in the transmission of LSDV between cattle. This notion is supported by experimental evidence obtained with Aedes aegypti mosquitoes [4], ixodid ticks (*Amblyomma hebraeum*, *Rhipicephalus appendiculatus* and *Rhipicephalus* (Boophilus) decoloratus) [5,6,7,8,9], biting flies (*Stomoxys calcitrans*) [10,11] and horseflies (*Haematopota* spp.) [10].

Stable flies (*Stomoxys calcitrans*) are of special interest as they have been shown to act as an efficient mechanical vector for several other infectious diseases and their high cosmopolitan abundance near cattle [12]. LSDV transmission by *Stomoxys calcitrans* has been observed during three independent animal experiments in our laboratory. During these studies, naïve acceptors developed typical LSDV skin nodules accompanied by a clear viremia and seroconversion after exposure to *S. calcitrans* which had fed upon donor bulls which had developed skin nodules after experimental inoculation [13].

Under experimental conditions, up to 50% of LSDV-infected animals do not develop the typical LSDV nodules and remain uninfected or subclinically infected [3,10,14,15]. Currently, no conclusive data are available regarding the role of these animals in LSDV epidemiology. This is an important knowledge gap and needs to be addressed in order to provide sufficient scientific information to decision-makers on how to handle animals with subclinical infections. This will support their control/eradication strategies and subsequent actions, such as the culling of all susceptible animals and/or mass vaccination [16]. One modelling study [3] suggested that subclinical cattle play little part in virus transmission relative to cattle with skin lesions due to a low probability of virus acquisition by feeding vectors. In the absence of experimental data and due to the variability in appearance and intensity of clinical symptoms, viremia, seroconversion and immunological responses after exposure to LSDV, we wanted to study this in vivo. Therefore, an animal trial was conducted whereby LSDV subclinically and preclinically infected cattle were used to feed *S. calcitrans* flies and to study the transmission by them to acceptor animals.

## 2. Materials and Methods

### 2.1. Virus Strain

The LSDV field isolate (LSD/OA3-Ts. MORAN, Washington, DC, USA; passage 4) used in this study was isolated from cattle during an outbreak in Israel in 2012. The isolate was grown in OA3.Ts cells as described by Babiuk et al. [17]. 

### 2.2. Vectors

Biting flies, more specifically *Stomoxys calcitrans*, were caught in the vicinity of a cattle herd (Drongen, Belgium) using insect nets and were subsequently identified using morphological keys [18]. Batches of 50–200 *S. calcitrans* were kept within plastic cages for an average of 2 days at room temperature (±20 °C) before being used in the experiment. During this period, they only received moist cotton pads without added sugar. The cages were self-made, oval shaped, covered at each end with a mosquito net (mesh size: 1 mm) sleeve and were approximately 4 cm × 15 cm × 7 cm (height × length × width) [10].

### 2.3. General Experimental Set-Up

Belgian LSDV free Holstein bulls (4–6 months old, *n* = 26) were split randomly in 13 donor and 13 acceptor animals. All animals were checked by PCR and by serological tests (IPMA) to verify their LSDV negative status before the experiment started. Cattle were allowed to acclimatize for 7 days before the onset of the trial. Donor and acceptor animals were kept separately in insect-free Biosafety Level 3 facilities (Sciensano, Machelen, Belgium). Water was available ad libitum and animals were fed once each day. Animal experiments were performed in accordance with the European Union and Belgian regulations on animal welfare in experimentation. The protocol was approved by the joined ethical committee of Sciensano, authorization number 20190211-01_Rev20190809.

### 2.4. Experimental Inoculation of Donor Animals 

A standardized challenge protocol [13] was used whereby donor animals are inoculated intravenously in the vena jugularis (5 mL) and intradermally in the neck (1 mL). The latter was conducted by injecting 250 μL in 2 different locations on both sides of the neck (4 in total). The concentration of the inoculum was 10^6.3^ TCID_50/mL_. The first day of inoculation of the donor animals was designated as 0-day post inoculation (0 dpi).

### 2.5. Disease Status of LSDV-Inoculated Animals 

The following definitions were used to allocate one of four following statutes to LSDV-inoculated animals dependent on the disease outcome: (1) Clinically infected animals are animals with typical LSDV skin nodules which have been confirmed by real-time PCR; (2) Preclinical LSDV animals are animals which have no skin nodules at the moment of manipulation (sampling, vector exposure…) but are or have been LSDV positive in real-time PCR in blood samples. These animals do develop skin nodules at a later stage of the trial and become thus clinically infected; (3) Subclinical animals are animals which never develop skin nodules during the complete duration of the animal trial but in which proof of productive virus replication has been found based on laboratory analysis, here only assessed by measuring viremia [19]; (4) Non-infected animals are animals which do not develop skin nodules but in which no proof of productive virus replication could be found by laboratory analyses. 

### 2.6. Placement and Feeding of S. calcitrans on Donor and Acceptor Cattle 

*S. calcitrans* were field caught and caged (50–200 flies per cage). On the day of transmission, the flies were allowed to feed for 10 min on LSDV preclinical or subclinical donor bulls. Following 1 h of rest, the flies were transferred to the acceptors and allowed to feed for 10 min. The placement of the flies on the donors and acceptors was done on the shoulders of the animals (this is more than 30 cm away from the inoculation site of the donor animals) [10]. An overview of the placement (days and number) of flies is given in Table 1. The first day of feeding of the *S. calcitrans* flies on the acceptor animals was designated as 0-day post feeding (0 dpf). After placement on the acceptors, all the insects were stored at −20 °C until use.

### 2.7. Clinical Scoring

Several clinical parameters were followed throughout the duration of the trial: fever, food uptake, swelling at inoculation site, number of skin nodules, location of skin nodules and prescapular lymph node swelling. These observations were translated to a clinical score as described in Haegeman et al. [13]. The onset of prolonged fever was defined as a body temperature of 39.3 °C or more for 2 or more consecutive days. 

### 2.8. Sample Collection 

Blood samples (EDTA, clotted and heparin) were collected at regular intervals during the complete duration of the trial. This was once during the acclimatization period; on the day of challenge (0 dpi; donors) or first vector exposure (0 dpf; acceptors); during the post-infection period, this was on a daily basis during the second week and twice in the first and third week; during the post-feeding period on acceptors, this was twice per week.

### 2.9. DNA Extraction and Real-Time PCR 

Viral DNA extraction from blood was carried out as described by Haegeman et al. [20]. The detection of capripox viral genome was carried out using a real-time PCR panel [20].

### 2.10. Serological Analysis

The serum samples collected during the trial were analyzed with the immunoperoxidase monolayer assay (IPMA) as described by Haegeman et al. [21]. In addition, the samples were also tested with a commercial ELISA (ID Screen ^®^CPV Double Antigen (IDVET, Montpellier, France)), according to the manufacturer’s instructions and the virus neutralization test. For the latter, this was performed according to the WOAH Terrestrial Manual [22], except for the visualization of the non-neutralized virus, which was carried out as described in Haegeman et al. [23]. 

### 2.11. IFNγ Release Assay

Heparin blood samples were directly used for analysis as described by Haegeman et al. [13]. Briefly, the blood (1.5 mL) was stimulated (100 µL/well) with LSDV virus (10^6.8^ TCID_50_/mL). 1× PBS (as measure for the background) or Pokeweed mitogen (160 µg/mL dissolved in 1× PBS), as a positive control. After overnight incubation, the plasma was collected and analyzed using the BOVIGAM^®^ 2G kit (Thermofisher; Merelbeke, Belgium). The delta OD is calculated as follows: OD value [sample]—OD value [PBS]. The cut-off of the sandwich ELISA for positivity was set to 0.3. The OD values of the positive and negative controls were monitored over time to identify false positive and negative results.

## 3. Result

### 3.1. Donor Animals

**Figure 1:**
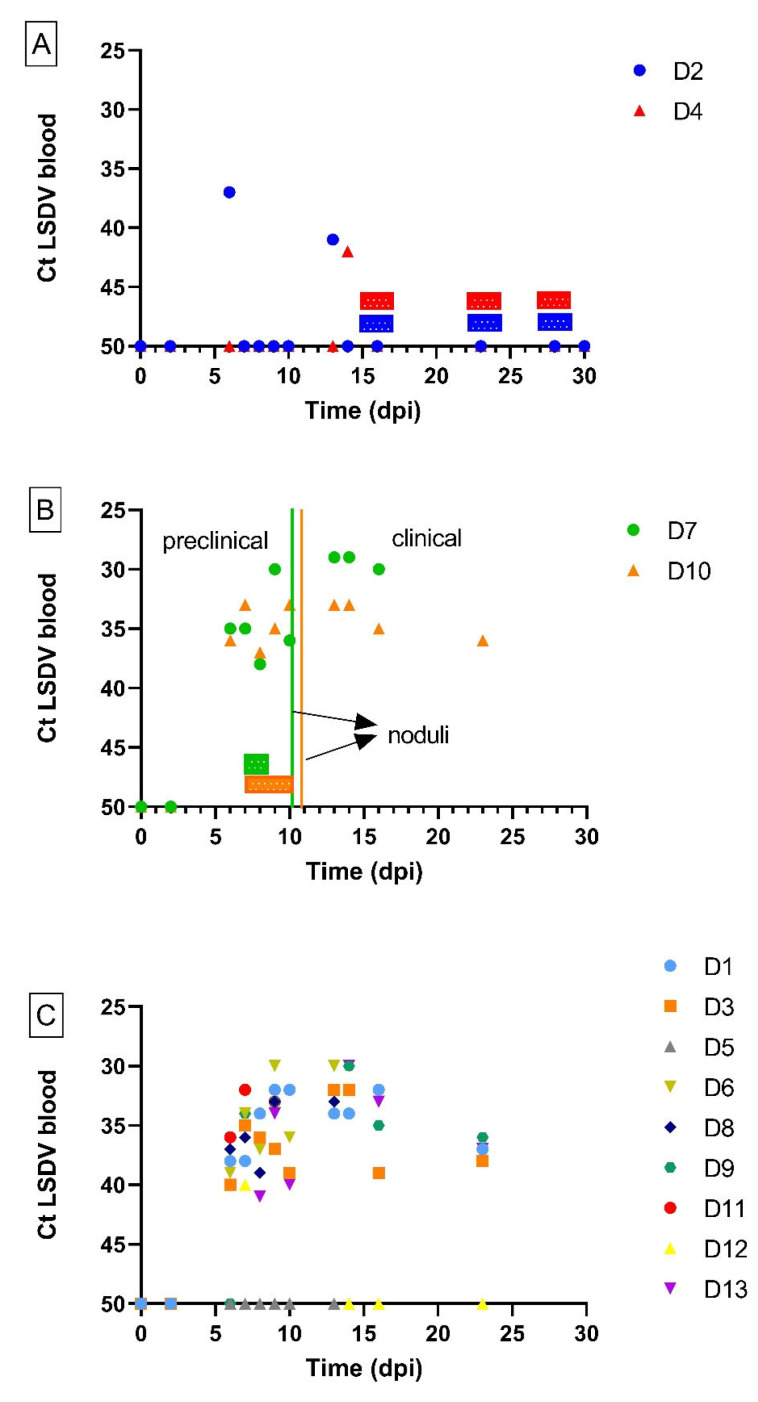
Detection of LSDV DNA by real-time PCR (Ct) in the blood of donor animals over time in subclinical (**A**) and preclinical (**B**) donor animals exposed to *S. calcitrans* flies and in non-exposed donor animals (**C**). Bars above the *X*-axis indicate the time periods during which *S. calcitrans* flies were placed for 10 min each day on infected donor animals.

**Figure 2:**
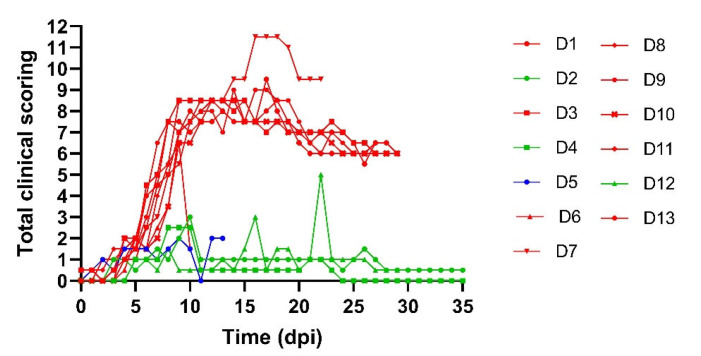
Total clinical scoring of donor animals based on several parameters: fever, food uptake, swelling at inoculation site, number of skin nodules, location of skin nodules and erythematous area. Animals with skin nodules (red), subclinically infected animals (green) and non-infected (blue).

All donor animals displayed a rise in body temperature around 6 dpi. This was either moderate and transient (maximum of 3 consecutive days; D2, D4 and D12) or evolved to an important fever period whereby animals had body temperatures above 40 °C for multiple days. Nine of the thirteen donor animals developed skin nodules, typical for LSDV (Table 2), and became viremic from 6 dpi onwards. The viremia remained until they were euthanized between 10 and 23 dpi (Figure 1). All, except D11, developed generalized infections with multiple characteristic skin nodules across the body. For D11, this was not the case, but this could be due to the fact that the animal was euthanized for ethical reasons at 10 dpi. Based upon the definitions of the LSDV infection status listed above, these animals were considered to be clinically infected. In contrast, donor animals D2, D4 and D12 never developed skin nodules while they were positive on real-time PCR for one to two days. Interestingly, these three animals were also the ones with a moderate and transient fever pattern. The final status of these donors was subclinically infected. One donor animal, D5 did not develop skin nodules and was never positive in the blood tested by real-time PCR. This animal was therefore considered non-infected. The total clinical scoring for the donor animals can be seen in Figure 2. A lower clinical score was observed for subclinically infected animals compared to animals which had developed skin nodules. The incubation period was defined as the time interval between the inoculation and the moment animals became viremic. This was found to be rather constant for all donor animals (6–7 days, except for donor animal D4 (14 days)). Seroconversion was seen for all donors (IPMA, Table 3), except for D5 and D11, but this could be due to the fact that these animals were euthanized earlier due to either ethical reasons (D11) or lack of viremia (D5).

### 3.2. Transmission from Preclinical Cattle 

Stable flies were fed upon D7 and D10 between 7 and 8 (D7) or 10 (D10) dpi when they were viremic but without skin noduli (Table 1 and Table 2). As both animals developed noduli on 10 (D7) and 11 (D10) dpi, the flies which had fed upon them were used to study the transmission from preclinically infected cattle. The donor animals were clearly positive for LSDV genome in the blood when the vectors were allowed to feed, as the cycle threshold (Ct) values varied between 33 and 38 (Figure 1).

No evidence of LSDV transmission by *Stomoxys calcitrans* was observed in any of the eight acceptor animals after exposure to flies fed on preclinical animals D7 and D10. This was supported by the absence of clinical symptoms (no fever, no skin nodules and no swelling of the prescapular lymph node), no viremia and no seroconversion in all these acceptor animals (A1–A8) (Table 4). Furthermore, no cellular immune response was induced, based on the absence of an IFNg release response upon stimulation of the heparin blood with LSDV.

### 3.3. Transmission from Subclinical Animals

Stable flies were placed upon subclinical animals D2 and D4 on multiple days (Table 1). As mentioned above, these donors never developed skin noduli but were or had been viremic. On the days of vector feeding (Figure 1), the Ct values of these donors were above 37 or negative in the blood. Consequently, these flies were used to study transmission from subclinical animals. 

**Figure 3:**
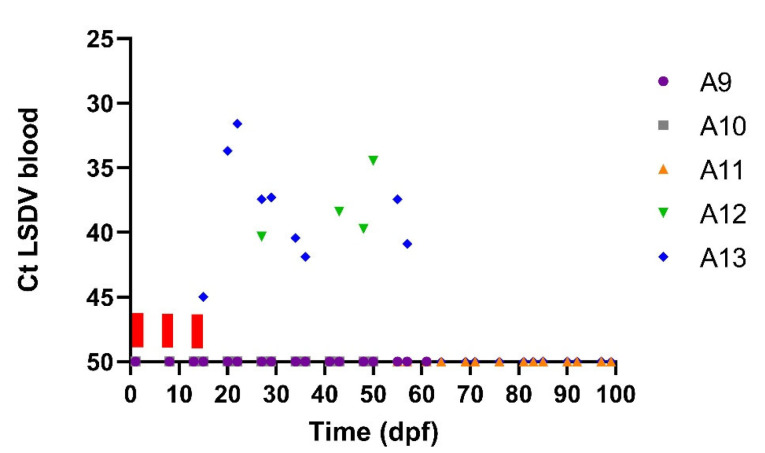
Detection of LSDV DNA by real-time PCR (Ct) in blood acceptor animals over time (dpf) after exposure to flies from subclinical animals D2 and D4. Bars above the *X*-axis indicate the time periods during which *S. calcitrans* flies were placed for 10 min each day on these acceptor animals.

**Figure 4:**
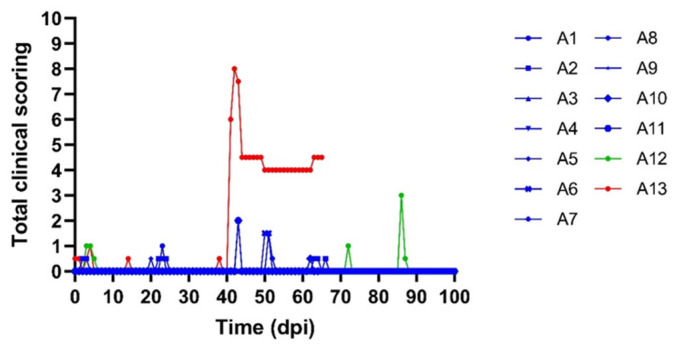
Total clinical scoring of acceptor animals. Animals with skin nodules (red), subclinical animals (green) and non-infected (blue).

One of the acceptors (A13) had a transient fever spike around 26/27 dpf while the body temperatures remained normal in the other four acceptor animals receiving the flies from the subclinical donors. The same animal, A13, also developed skin nodules. The productive LSDV infection status of A13 was confirmed by lab analyses. A clear viremia was observed for A13 from 20 to 36 dpf. Interestingly, viremia was also observed for A12. The blood of this animal was positive on a real-time PCR at 27 dpf and from 43 to 57 dpf (Figure 3). As A12 did not develop skin nodules, the infection was classified as subclinical. The peak Ct values varied between 31 (A13) and 34 (A12) (Figure 3) for these two acceptor animals while the peak Ct value from the subclinical donor animal (D4) on which the *S. calcitrans* flies were fed was only 42 (Figure 1). Since flies were fed several times on donors and acceptors and the exact occasion at which transmission occurred remains unknown, the incubation period in the acceptor animals cannot be exactly determined and is between 6 and 27 days (Table 4). In Figure 4, it can be seen that the clinical score of acceptor animal A13 is clearly higher than in the other animals that stayed subclinical (A12) or did not develop LSD. 

**Figure 5:**
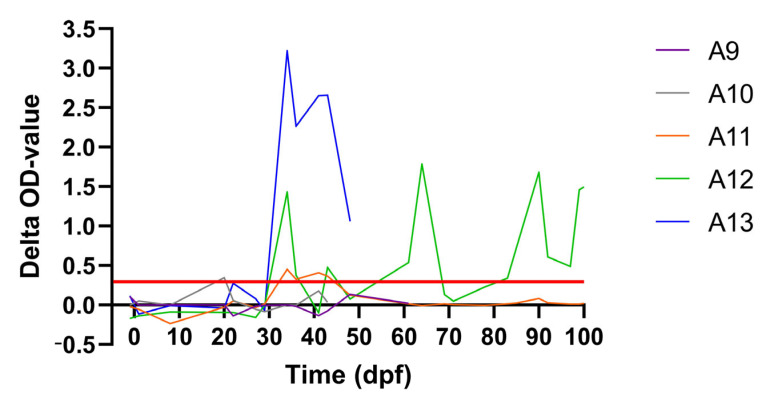
The IFNg response after stimulation of the heparin blood of the acceptor animals exposed to stable flies from subclinical animals. Red line: cut-off for positivity.

The transmission of LSDV from the subclinical donor to the acceptor animals A12 and A13 is further supported by the observed seroconversion, as determined by the IPMA and ELISA (Table 4), and the induced IFNg response (Figure 5). Seroconversion by IPMA was also detected in A9, albeit very weak and temporary. No confirmation of this seroconversion was seen with ELISA and no IFNg response was observed during the complete duration of the trial. Furthermore, no proof of productive virus replication (viremia) was found with real-time PCR. 

## 4. Discussion

This is the first time the role of subclinical and preclinical LSDV-infected cattle as a source of LSDV transmission has been studied in vivo. The outcome of the experimental inoculation to produce LSDV-infected donor animals was comparable to previous animal experiments [10,19]. Approximately 23% of the donors (3 out of 13) displayed a subclinical form of the disease, which is a significant part of the inoculated group. In contrast to the animals with skin nodules, the viremia in subclinical donor animals is low, and skin lesions are absent in these animals. Nevertheless, it was shown that *S. calcitrans* flies were able to transmit LSDV from these subclinical LSDV-infected donor animals to two out of five naïve acceptor animals. This transmission is supported by the development of viremia, seroconversion and induction of an IFNg response. Interestingly, one of these two acceptors developed the typical LSDV skin nodules while the other did not. Based upon the formulated definitions, this second acceptor is considered to be subclinically infected [19]. As no skin nodules are present on the subclinical donors, the virus needs to have been acquired by the flies during feeding on normal looking skin containing LSDV. A third acceptor animal, A9, was a special case as a temporary and very weak seroconversion was seen on IPMA between 27 and 43 dpf (returned negative on 50 dpf). However, this was not supported by the ELISA results and no IFNg response was observed after stimulation with the antigen. No viremia was observed in the blood but as there was no daily sampling, it could have been missed if it was very short. Therefore, a subclinical infection cannot be 100 percent excluded, but without further proof, we did not give a final clinical status to this animal. 

Viral DNA was already detected in biopsy samples of normal skin from subclinical animals in two other studies [3,19], and *S. calcitrans* flies were found to be LSDV positive through PCR two days post-feeding on subclinical cattle [3]. Additionally, a study with ticks (*R. microplus* females) demonstrated that feeding on viremic cattle without showing multiple skin lesions resulted in ticks being RT-PCR positive [24]. Our results are also in line with studies on other vector-borne diseases whereby asymptomatic individuals may be an important source of the pathogen for vectors and may help to maintain the transmission cycle. For instance, asymptomatic wild boars or pigs (non-vaccinated) can be a reservoir for Japanese encephalitis virus [25]. Similarly, cattle are usually asymptomatic carriers after being infected with BTV, which leads to BTV spreading easily in the herd, mainly through the bites of biological vectors, such as Culicoides [26]. 

This could also be the case for LSD as animals without clinical symptoms go unnoticed in the field and are not separated from naive animals. Our data thus indicate that the role of subclinically infected animals in LSDV transmission should not be neglected and that they could contribute more to virus transmission than previously thought. Although they are probably not the drivers of LSDV outbreaks, they probably provide an additional way for the disease to jump to other geographical locations next to be spread by vectors and illegal transport. This could occur through the transport of non-clinical animals to the slaughterhouse or to other farms.

In the preclinical donor animals in this study, a higher genomic load was observed compared to the subclinical donor animals. Nonetheless, no acceptor animals became LSDV infected by *S. calcitrans,* which had fed upon these preclinical LSDV-infected donor animals. Currently, no clear explanation can be given for this lack of transmission. Firstly, it cannot be excluded that this lack of transmission was due to insufficient number of acceptor animals, it needs to be mentioned that more acceptors were exposed to flies from preclinical than subclinical donors. Furthermore, no detectable antibodies were found in the serum of the preclinical donors at the moment of vector feeding, making the lack of infectious virus due to antibody neutralization (while still being PCR positive) rather unlikely. In addition, the feeding of the flies on subclinical donors was even on a later time point than those that fed on preclinical donor, making complete neutralization of the virus by antibodies even more unlikely. We also determined viremia using venous blood, while the flies were fed on the skin. The amount of (live) virus present in the skin (if any) could have been less than presumed based on the Ct values of the venous blood. This in turn could explain the lack of transmission observed in this study. However, not much is known on the different virus kinetics in the skin and venous blood following infection (experimental or natural) and forms an important knowledge gap especially relating to transmission risks. Although we did not find proof of LSDV transmission from vectors fed on preclinical donors, the number of animals tested remains limited and this should be studied in more detail before concluding that this is not possible.

Due to the experimental setup used in this study, it cannot be ascertained how many LSDV-infected flies continued their blood meal on the acceptor animals and how many viruses were transmitted to the acceptors. The incubation period in the inoculated donors was, in general, stable (5–7 days), except for one subclinical infection (14 days). In contrast, much more variability was seen in the acceptors with a minimum of 6 to 13 days but could go up to 27 days. No confirmation can be given of the exact incubation period due to the experimental setup with multiple periods of feedings per animal. Furthermore, this variation in the length of the incubation period in acceptors is probably related to the inoculated viral dose. This was also seen in a previous in vivo LSDV transmission study with clinically infected donors (10), where the incubation period ranged between 6 and 26 days. 

The vectors were placed on the shoulder region of the donor (and acceptor) animals and were more than 30 cm separated from the intradermal (side of the neck) and intravenous (jugular vein in the jugular furrow) injection sites. This implicates that the virus that was taken up by the stable fly during feeding cannot be the inoculum itself that was injected at those sites but rather the disseminated virus. However, the impact of intravenous inoculation on the latter is unknown and needs further research as “natural” infection by vectors is intradermal.

Our results strongly support that the vaccination of all animals is the most effective control measure to prevent the spread of LSDV, as complete stamping out of all potentially infected animals (including subclinical cattle) is often not supported for economic reasons [27] or is not allowed due to specific regulations against cattle slaughter, such as in India [28]. This is in line with the EFSA report 2022, which states that according to a model for the transmission of LSDV between farms, vaccination has a greater effect in reducing LSDV spread compared to any culling policy, even when low vaccine effectiveness is considered [29]. This is further supported by the fact that the identification of subclinical animals remains very difficult as no skin nodules develop, and the viremia is often very weak and short, which means that it can be easily missed upon sampling. Additional research is needed to further characterize and identify these types of animals. 

Another control measure that is often overlooked is vector control on the animals themselves, which could have a great impact. Furthermore, in affected countries, the seasonal movement of cattle could be recommended as seasonal variation in LSD infection is possibly associated with the relative abundance of the stable fly, *Stomoxys calcitrans* [30].

## 5. Conclusions

In summary, our findings demonstrate the contribution of subclinically infected cattle to the transmission of Lumpy Skin Disease virus by *Stomoxys calcitrans*. The latter has implications for the control policies implemented by decision-makers and show that subclinical animals should be taken into account when modeling the risk of LSDV transmission.

## Figures and Tables

**Table 1:** Overview of placement *S. calcitrans* on the donors, including the exact number of stable flies used and the moment of attempted transmission. #: number of *S. calcitrans*. dpi: days post inoculation; Dx: Donor Animal x; Ax: Acceptor animal x.

#	dpi		Donor Animals												Acceptor Animals												
		D1	D2	D3	D4	D5	D6	D7	D8	D9	D10	D11	D12	D13	A1	A2	A3	A4	A5	A6	A7	A8	A9	A10	A11	A12	A13
600	7–8–9–10										600								200	200		200					
300	8–9–10										300								100	100		100					
300	7–8							300								100	100	100									
600	8							600								200	200	200									
380	10										380				160					60	160						
200	15–16–17		200																				100	100			
300	15–16–17				300																				100	100	100
180	22–23–24		180																				90	90			
270	22–23–24				270																				90	90	90
300	27–28–29		300																				100	100	100		
200	27–28–29				200																					100	100
3630																											

**Table 2:** Detection of LSD DNA by real-time PCR in blood and skin nodules, IPMA results and accorded clinical status to donor animals; † euthanasia.

ID Donor Animal	Peak Ct in Blood (dpi)	Viremic Period (dpi)	Noduli (dpi): FIRST Appearance (Generalization)	Ct Nodules on 9/10 dpi	IPMA	Clinical Diagnosis
D1	31.80 (9)	6 until 23 †	7 (10)	18.32	pos	clinical
D2	37.48 (6)	6 and 13	none	/	pos	subclinical
D3	31.56 (14)	6 until 23 †	6 (9)	31.99	pos	clinical
D4	41.99 (14)	14	none	/	pos	subclinical
D5	/	/	none	/	neg	non-infected
D6	29.75 (13)	6 until 13 †	9 (10)	21.8	pos	clinical
D7	28.61 (13)	6 until 16 †	10 (10)	13.45	pos	clinical
D8	32.02 (10)	6 until 13 †	7 (9)	14.72	pos	clinical
D9	30.06 (14)	7 until 23 †	6 (7)	13.98	pos	clinical
D10	32.96 (14)	6 until 23 †	11 (13)	19.59	pos	clinical
D11	31.80 (7)	6 until 10 †	6 (/)	†	neg	clinical
D12	40.20 (7)	7	none	/	pos	subclinical
D13	30.23 (14)	6 until 23 †	6 (9)	14.82	pos	clinical

**Table 3:** The immunoperoxidase monolayer assay (IPMA) scoring for all donor animals. The IPMA scoring is expressed as strong positive, positive or weak positive as indicated with the color code shown at the bottom of the table.

	D1		D2		D3		D4		D5		D6		D7		D8		D9		D10		D11		D12		D13	
	1/50	1/300	1/50	1/300	1/50	1/300	1/50	1/300	1/50	1/300	1/50	1/300	1/50	1/300	1/50	1/300	1/50	1/300	1/50	1/300	1/50	1/300	1/50	1/300	1/50	1/300
0 dpi	N	N	N	N	N	N	N	N	N	N	N	N	N	N	N	N	N	N	N	N	N	N	N	N	N	N
2 dpi	N	N	N	N	N	N	N	N	N	N	N	N	N	N	N	N	N	N	N	N	N	N	N	N	N	N
6 dpi	N	N	N	N	N	N	N	N	N	N	N	N	N	N	N	N	N	N	N	N	N	N	N	N	N	N
7 dpi	N	N	N	N	N	N	N	N	N	N	N	N	N	N	N	N	N	N	N	N	N	N	N	N	N	N
8 dpi	N	N	N	N	N	N	N	N	N	N	N	N	N	N	N	N	N	N	N	N	N	N	N	N	N	N
9 dpi	P	N	N	N	N	N	N	N	N	N	P	N	N	N	N	N	N	N	N	N	N	N	P	N	P	neg
10 dpi	P	P	P	N	N	N	N	N	N	N	P	N	N	N	N	N	N	N	N	N	N	N	P	P	P	P
13 dpi	P	P	P	N	P	N	P	N	N	N	P	P	P	P	P	N	P	N	P	N	†	†	P	P	P	P
14 dpi	P	P	P	N	P	P	P	N	†	†	†	†	P	P	†	†	P	N	P	P	†	†	P	P	P	P
16 dpi	P	P	P	P	P	P	P	N	†	†	†	†	P	P	†	†	P	N	P	P	†	†	P	P	P	P
23 dpi	P	P	P	P	P	P	P	N	†	†	†	†	†	†	†	†	P	P	P	P	†	†	P	P	P	P
28 dpi	†	†	P	P	P	P	P	N	†	†	†	†	†	†	†	†	†	†	†	†	†	†	†	†	†	†
30 dpi	†	†	P	P	†	†	P	N	†	†	†	†	†	†	†	†	†	†	†	†	†	†	†	†	†	†
36 dpi	†	†	P	P	†	†	P	N	†	†	†	†	†	†	†	†	†	†	†	†	†	†	†	†	†	†
	P	Strong Positive			P	Positive			P	Weak Positive			N	Negative				†	Euthanasia							

**Table 4:** Detection of LSD DNA by real-time PCR in blood, incubation period, days of viremia, IPMA, ELISA and clinical diagnosis of the acceptor animals.

ID Acceptor Animal	Fed on Donor Animal	First Day Flies on (dpi)	Incubation Period: Min-Max (Days) *	Peak Ct in Blood (dpf)	Viraemic Period (dpf)	Nodules Present	IPMA	ELISA	Clinical Diagnosis
A1	preclinical D10	10 dpi	/	/	/	no	neg	neg	non-infected
A2	preclinical D7	7 dpi	/	/	/	no	neg	neg	non-infected
A3	preclinical D7	7 dpi	/	/	/	no	neg	neg	non-infected
A4	preclinical D7	7 dpi	/	/	/	no	neg	neg	non-infected
A5	preclinical D10	7 dpi	/	/	/	no	neg	neg	non-infected
A6	preclinical D10	7 dpi	/	/	/	no	neg	neg	non-infected
A7	preclinical D10	10 dpi	/	/	/	no	neg	neg	non-infected
A8	preclinical D10	7 dpi	/	/	/	no	neg	neg	non-infected
A9	subclinical D2	15 dpi	/	/	/	no	pos	neg	undetermined
A10	subclinical D2	15 dpi	/	/	/	no	neg	neg	non-infected
A11	subclinical D2–D4	15 dpi	/	/	/	no	neg	neg	non-infected
A12	subclinical D4	15 dpi	13–27	34.44 (50)	27, 43 until 57	no	pos	pos	subclinical
A13	subclinical D4	15 dpi	6–20	31.59 (22)	20 until 36	yes	pos	pos	clinical

* Since flies were fed several times on donors and acceptors and the exact occasion at which transmission occurred remains unknown, the incubation period in the acceptor animals is indicated by minimum or maximum number of days.

## Data Availability

Not applicable.
